# Health-related quality of life 5 years after carpal tunnel release among patients with diabetes: a prospective study with matched controls

**DOI:** 10.1186/1472-6823-14-85

**Published:** 2014-10-17

**Authors:** Niels OB Thomsen, Jonas Björk, Ragnhild I Cederlund

**Affiliations:** Department of Clinical Sciences Malmö - Hand Surgery, Lund University and Skåne University Hospital, Malmö, Sweden; Competence Centre for Clinical Research, Lund University and Skåne University Hospital, Lund, Sweden; Department of Health Sciences, The Vårdal Institute, Lund University, Lund, Sweden

**Keywords:** Carpal tunnel syndrome, Diabetes, Health-related quality of life, Sense of coherence, SF-36

## Abstract

**Background:**

Carpal tunnel syndrome (CTS) is the most common entrapment neuropathy encountered in diabetes. The short-term improvement after carpal tunnel release has previously been demonstrated not to differ between patients with and without diabetes, despite a marked impairment in health-related quality of life (HRQL) among the former. In this study, we compare HRQL 5 years after carpal tunnel release between these two groups of patients.

**Methods:**

In a prospective series, 35 patients with diabetes and CTS were matched with 31 control patients with idiopathic CTS but no diabetes. At the 5-year follow-up patients completed the Medical Outcomes Short-Form 36 (SF-36) and Antonovsky’s sense of coherence (SOC) questionnaire. Differences in changes over time were compared between patients with and without diabetes using mixed model analysis.

**Results:**

Although patients with diabetes reported a significant decrease in physical functioning (p =0.004) as compared to patients without diabetes, postoperative improvement was maintained in the physical domains, role physical and bodily pain. A more pronounced decline in the mental health domain, social function (p =0.03), was demonstrated among patients with diabetes. There was no evidence of any difference in SOC between the patient groups.

**Conclusion:**

Patients with diabetes retained their improvement in physical domains sensitive to changes after carpal tunnel release in the long-term, despite a decline in other domains of both physical and mental HRQL. This differed from patients without diabetes. Differences in SOC could not explain the sharper decline in these domains among patients with diabetes.

## Background

Carpal tunnel syndrome (CTS), the most common entrapment neuropathy in the upper extremity is estimated to occur in 3% of the general population [1]. In patients with diabetes the prevalence of CTS has been anticipated to be approximately 15%, and increasing up to 30% for those with diabetic peripheral neuropathy [2]. The reason why CTS is more common in diabetes is not fully clarified. It has been suggested that a nerve, with already established endoneurial hypoxia caused by diabetes, could be more vulnerable to local compression [3]. Additional mechanisms may involve increased concentrations of advanced glycation end products as well as myofibroblasts leading to synovitis and stiffening of the collagen tissue [4].

Diabetes adversely affects health-related quality of life (HLQL). An even larger impact on HRQL is related to poor metabolic control, diabetic complications such as peripheral neuropathy, and the development of psychiatric symptoms [5–7].

We have previously published 1-year follow-up results on carpal tunnel release among age- and gender-matched patients with and without diabetes [8, 9]. We evaluated HLQL in terms of the generic Medical Outcomes Short-Form 36 (SF-36), and demonstrated that improvement in the physical quality of life after surgery did not differ between the two groups, although patients with diabetes did not reach population norm values. In contrast, a decline in the mental quality of life was found. Long-term HRQL evaluation after carpal tunnel release in patients with diabetes has not been explored to date.

Sense of coherence (SOC) refers to how one person’s perception, attitude, and response to stressful situations influences their capacity to be and remain healthy [10]. An association has been demonstrated between low SOC, diabetes and poor glycaemic control [11]. A strong SOC would suggest increased coping resources to enhance self-care behaviour and prevent further diabetic complications [12, 13].

The purpose of this investigation was to extend our previously published clinical trial, to compare HRQL 5 years after carpal tunnel release among patients with and without diabetes. Adding the SOC questionnaire to the long-term follow-up, allowed us to assess a possible association between SOC, diabetes and HRQL.

## Methods

After approval by the Regional Ethical Review Board, Lund University, 35 consecutive patients who attended our outpatient clinic (2004 to 2007) with type 1 or type 2 diabetes, and who had had CTS for at least six months, were enrolled in the study. We tried to age- and gender-match each patient with diabetes with a control patient with idiopathic CTS but no diabetes. We allowed a maximum age difference of 5 years. In total, 31 control patients were identified during the enrolment. The CTS diagnosis was based on a characteristic clinical history, symptoms and verified by a nerve conduction study. We have previously published a detailed description of the eligibility criteria and the surgical procedure [8]. In the present extended study, performed from 2010 to 2012, patients included in the follow-up had undergone surgery at least 5 years previously. Written informed consent was obtained from all patients.

### Study population

Two patients with diabetes were unable to attend the present study owing to other forms of illness, and one patient with idiopathic CTS declined to participate. Thus the study group comprised of 33 patients with diabetes, and 30 patients without diabetes having CTS. Thereby, the overall long-term attendance rate is 95%.

### Electrophysiology

Median nerve conduction studies were performed using a Viking Select electromyograph (Viasys Inc. Madison, WI, USA). The investigations were conducted with surface electrodes (skin temperature kept above 30°C). The diagnosis of CTS was based on measurement of a reduced antidromic sensory conduction velocity, over the carpal tunnel segment (<44 m/s) and a prolonged distal motor latency (>4.1 ms) from the wrist to the abductor pollicis brevis muscle [14].

### Measurement of HbA1c

The level of HbA1c was measured by immunoassay from a venous blood sample collected on the same day as the questionnaires were filled out at baseline and the 5-year follow-up.

### Outcome questionnaire SF-36

The Medical Outcomes SF-36 is a generic HRQL questionnaire [15, 16]. It measures eight health domains divided into a physical component score (physical functioning, role limitations because of physical health problems, bodily pain, general health perceptions) and a mental health component score (vitality, social functioning, role limitations because of emotional problems, and general mental health). Responses within each domain are translated into a score ranging from 0 (poor health) to 100 (optimal health). To compare patients’ SF-36 health profile to that of the general population, we calculated SF-36 population norms based on 924 age- and gender-matched subjects, who were chosen randomly from the Swedish database for population norms [16].

### Sense of coherence

According to Antonovsky, the coping resources of an individual comprise three core components: comprehensibility, manageability, and meaningfulness [10]. To assess these 3 aspects we used Antonovsky’s 13-item SOC scale. The total score ranges from 13 to 91, where each item has a score from 1 (never) to 7 (very often). A higher score indicates a stronger SOC.

### Statistical analysis

Both HRQL (SF-36) and SOC were assessed prior to the operation (baseline) and at the 1 and 5 years follow-up. Differences in SF-36 and SOC between the groups at these three measuring points were tested using the two-tailed Mann–Whitney *U*-test. Correlation analysis between the two rating scales was performed using the Spearman rank correlation coefficient (r_s_).

For the repeated measurement of SF-36, differences in changes over time from baseline to 1 year and from baseline to 5 years, were compared between patients with and without diabetes using mixed model analysis with an autoregressive model of order one for the residuals. The differences compared to baseline, for each SF-36 domain, were modeled with diabetes, gender, and time (1 year and 5 years) as fixed effect categorical variables, with age and baseline SF-36 measurements as fixed effect continuous covariates and with a random effect for each patient.

Statistical analysis was performed using SPSS 20.0 for Windows (SPSS Inc., Chicago, IL, USA). P <0.05 was considered statistically significant.

## Results

The demographic and clinical characteristics of the patients are shown in Table 1. The duration of CTS was significantly shorter in patients without diabetes (p ≤0.008) Patients with diabetes (15 type 1 and 20 type 2) had a significantly higher HbA1c level (p ≤0.001) but overall demonstrated good glycaemic control. Indicating of more pronounced nerve pathology, the patients with diabetes had a significantly lower median nerve sensory conduction velocity (p ≤0.03) and a prolonged distal motor latency (p ≤0.003) than the patients without diabetes.

**Table 1:** **Patient characteristics at baseline and 5-year follow-up**

Characteristics*	Patients with diabetes	Patients without diabetes
**Baseline**	(n =35)	(n =31)
Age (years)	54 (31–73)	51 (35–77)
Female/Male (n)	22/13	19/12
Duration of CTS (months)	24 (8–96)	36 (12–180)
Duration of diabetes (years)	15 (1–43)	–
BMI (kg/m^2^)	28.1 (18.8 – 36.7)	26.3 (20–36.2)
HbA1c (mmol/mol)	50 (29 – 85)	25 (18 – 32)
HbA1c (%)	6.7 (4.8 – 9.9)	4.4 (3.8 – 5.1)
Median nerve, SNCV	20 (0–39)	25 (0–35)
Median nerve, DML	5.9 (3.8-10.0)	5.2 (3.3-8.4)
**Follow-up, 5 years**	(n =27)	(n =30)
Baseline to Follow-up (months)	68 (59 – 82)	64 (59–69)
BMI (kg/m^2^)	28.7 (17 – 38.1)	25.7 (20.4 – 40.8)
HbA1c (mmol/mol)	53 (38 – 91)	35 (23 – 49)
HbA1c (%)	7 (5.6 – 10.5)	5.4 (4.3 – 6.6)

*Values are median (range), unless otherwise stated.

BMI, body mass index; CTS, carpal tunnel syndrome; DML, distal motor latency (normal < 4.1 ms); HbA1c, glycosylated hemoglobin; SNCV sensory conduction velocity in the carpal tunnel segment (normal > 44 m/s).

### SF-36

At baseline, the physical component score and its four domains showed significant impairment in the patients with diabetes compared to the controls (p ≤0.05) (Table 2). Compared to population norms, impairment was most pronounced for the domains, bodily pain and role physical. With the exception of physical functioning, which had deteriorated for patients with diabetes between the 1-year and 5 years follow-ups, no difference in changes over time between the two groups could be shown in mixed model analysis, after adjustment for gender, age and baseline value of the domain. Bodily pain and role physical maintained their improvement at the 5-year follow-up. However, apart-from bodily pain, the physical component score (p =0.04) and those of its domains (p ≤0.03) were still significantly reduced for the patients with diabetes, compared to the patients without diabetes.

**Table 2:** **Medical Outcomes Short-Form 36 (SF-36)**

SF-36 domain	(Population norm [SD])		Difference in changes over time ^†^	
	Mean patient scores (SD)			
	Patients with diabetes (n = 35/33)*	Patients without diabetes (n = 31/30)*	Mean (95% CI)	p value
**Physical component score**	(49 [(10.0])			
Baseline	39 (7.4)	48 (9.0)		
1 year	44 (11.4)	49 (9.4)	-1.0 (-5.9 - 4.0)	>0.30
5 years	41 (14.9)	48 (10.9)	2.4 (-2.5 - 7.4)	>0.30
*Physical Functioning*	(84 [(21.6])			
Baseline	75 (17.8)	83 (16.5)		
1 year	79 (23.6)	87 (15.9)	2.9 (-5.8 - 11.5)	>0.30
5 years	67 (30.9)	84 (21.2)	12.8 (4.1 - 21.4)	0.004
*Role Physical*	(79 [35.1])			
Baseline	59 (36.9)	74 (38.4)		
1 year	64 (38.0)	85 (29.4)	15.1 (-1.2 - 31.4)	0.07
5 years	66 (41.4)	84 (35.0)	12.2 (-4.2 - 28.6)	0.14
*Bodily Pain*	(72 [27.1])			
Baseline	45 (22.7)	64 (28.0)		
1 year	64 (30.7)	71 (29.2)	-3.7 (-18.7 - 11.3)	>0.30
5 years	66 (32.5)	71 (28.9)	-4.9 (-19.9 - 10.1)	>0.30
*General Health*	(72 [23.2])			
Baseline	63 (22.7)	82 (20.1)		
1 year	61 (26.4)	82 (19.4)	-0.9 (-7.3 - 5.5)	>0.30
5 years	61 (30.1)	80 (21.4)	5.4 (-1.0 - 11.9)	0.10
**Mental component score**	(50 [11.0])			
Baseline	51 (9.2)	51 (8.1)		
1 year	46 (14.1)	53 (5.6)	6.8 (2.0 - 11.5)	0.006
5 years	49 (11.8)	52 (7.8)	3.2 (-1.7 - 8.0)	0.20
*Vitality*	(68 [24.2])			
Baseline	60 (23.7)	64 (24.2)		
1 year	62 (29.6)	72 (20.8)	6.3 (-4.6 - 17.3)	0.25
5 years	57 (30.9)	72 (24.3)	10.6 (-0.4 - 21.7)	0.06
*Social functioning*	(88 [21.4])			
Baseline	91 (15.1)	92 (14.7)		
1 year	79 (31.8)	94 (11.1)	14.3 (3.1 - 25.4)	0.01
5 years	76 (28.7)	90 (16.9)	12.6 (1.4 - 23.9)	0.03
*Role Emotional*	(83 [31.7])			
Baseline	82 (33.8)	82 (29.6)		
1 year	67 (43.3)	92 (14.2)	25.1 (10.7 - 39.4)	0.001
5 years	82 (33.4)	88 (28.3)	7.5 (-7.0 - 22.0)	>0.30
*Mental Health*	(80 [20.5])			
Baseline	77 (18.6)	79 (18.4)		
1 year	75 (24.1)	84 (14.4)	6.4 (-1.9 - 14.6)	0.13
5 years	78 (18.0)	84 (17.3)	5.2 (-3.1 - 13.6)	0.22

CI, confidence interval.

*Number of participants at baseline and 1-year/5-year follow-up.

^†^Differences in changes over time (non-diabetic minus diabetic patients) in a mixed model with adjustment for gender, age and baseline value of the domain.

At baseline, no significant differences could be shown between the two groups regarding the mental health component score or those of its domains (p ≥0.35) (Table 2). All scores were comparable to population norms. For the patients with diabetes, mixed model analysis at 1 year revealed a significant deterioration over time in the mental health component score, social functioning, and role emotional. At the 5-year follow-up, the decline was continuous for social functioning (p =0.03), while the mental health component score and role emotional had improved. Additionally, an almost significant (p =0.06) difference in change over time was observed between the groups for vitality at 5 years, due to a continuous decline for the patients with diabetes.

### Sense of coherence

At the 5-year follow-up, there was no significant difference in SOC (median [range]) between patients with diabetes (74 [42–86]) and patients without diabetes (74.5 [49–89]). A clearly significant correlation was demonstrated between SOC and SF36’s mental health component score r_s_ =0.68 (p <0.001) for the patients without diabetes, while it was almost significant for patients with diabetes, r_s_ =0.38 (p =0.054). No statistically significant correlations were demonstrated between SOC and the physical component score; in patients without diabetes r_s_ =0.26 (p =0.17) and patients with diabetes r_s_ =0.05 (p =0.82).

### Type 1 vs. type 2 diabetes

At baseline, patients with type 1 diabetes were significantly younger (years [range]) (43 [33–67] vs. 61 [31–73]; p =0,005), had a higher HbA1c (%) (7.3 [6.1-9.9] vs. 6.3 [4.8-9.0]; p =0.006), a lower BMI (kg/m^2^) (22 [19–33] vs. 31 [23–37]; p =0.001) and had had diabetes longer (27 [6–43] vs.10 [1–25]; p =0.001) than to those with type 2 diabetes. Nerve conduction study results were comparable between the two patient groups.

Neither at baseline nor at the 1-year follow-up could significant differences between patients with type 1 and those with type 2 diabetes be demonstrated for any of the SF-36 domains (data not shown). However, at the 5-year follow-up, physical functioning was significantly (mean [SD]) lower for type 2 (56 [31]) than for type 1 diabetes patients (79 [26]); p =0.03.

At the 5-year follow-up, there was no significant difference in SOC (median [range]) between patients with type 1 (68 [42–86]) and patients with type 2 diabetes (64 [51–84]; p =0.94.

### Carpal tunnel symptom complications

None of the patients demonstrated recurrent symptoms (numbness and parasthesias, with or without pain in the median nerve innervated fingers) or required re-operation for CTS.

## Discussion

HRQL for patients with diabetes has been shown to significantly decrease over a 5-year period, compared to otherwise healthy patients [17]. This decline has been attributed to the psychological and physical burdens of the disease and its complications. Patients with diabetes and diabetic hand symptoms (limited joint mobility, CTS, flexor tenosynovitis and Dupuytren’s contracture) were documented as having impaired physical health when compared to the norms for the general population [18]. Following the natural history of this patient group over a two-year period, revealed no significant change in HRQL.

This is the first report on HRQL in patients with diabetes, based on a follow-up of a minimum of 5-years, after carpal tunnel release. The domains bodily pain and role physical have previously been proved reliable, and sensitive in detecting changes after carpal tunnel release [19, 20]. In accordance, we were able to demonstrate a marked improvement in both role physical and bodily pain after surgery. As this improvement was maintained for both patients groups at the 5-year follow-up, we consider that the marked reduction in physical functioning in the patients with diabetes is caused by disorders other than CTS.

At the 1-year follow-up several of the mental health domain scores, had markedly deteriorated over time for patients with diabetes, compared to patients without diabetes. In this extended follow-up we therefore added the SOC questionnaire in order to investigate whether reduced coping resources could explain this difference. It has previously been demonstrated that SOC influences the outcome after major hand injuries [21]. For patients with diabetes a lower SOC score is associated with poorer glycemic control, and thus increased risk of diabetic complications [12]. However, we found no empirical evidence for difference in SOC between the two patient groups. This may imply that other factors, such as diabetes co-morbidity or complications, may influence the differing decline in the HRQL domains observed in the two patient groups.

One limitation of our study is the sample size, which may hamper our ability to detect important differences in outcome. The strengths of the study lie in the prospective design with matched controls and the high attendance rate of enrolled patients. The aim of this study was to evaluate the outcome of carpal tunnel release in patients with diabetes. However, it is evident that the long-term results are also influenced by the natural course of diabetes. Chronic macro- and micro-vascular diabetes complications are among the strongest determinants for HRQL and affect subjects with types 1 and 2 diabetes, differently [22]. We acknowledge the limitation of not being able to provide information about such complications. In addition, we did not include questionnaires on psychological factors such as anxiety or depression, which is established determinants of HRQL. We also combined results for patients with types 1 and 2 diabetes. However, it is somewhat reassuring that no notable differences in SF-36 domains were demonstrated at any point apart from for physical functioning at 5 years being lower for type 2 than for type 1 diabetes. It could be considered a limitation that we only assessed SOC at the 5-year follow-up. However, Antonovsky described SOC as a disposition rather than a personality trait. He considered SOC to be more or less stable once young adult hood was reached [10, 21]. We would, therefore not expect notable changes in SOC during the follow-up period. Finally, all patient were recruited from our local hospital area, where strict eligibility criteria were used to create two homogeneous patient groups based on CTS. All patient were Caucasians, but we cannot provide information on educational or socioeconomic status. Therefore, the generalizability of our results is limited to the setting of our study.

## Conclusions

Patients with diabetes retained their improvement in those physical domains sensitive to changes after carpal tunnel release in the long-term, despite a decline in other domains of physical and mental HRQL. Differences in SOC could not explain the sharper decline in these domains among patients with diabetes.

## Abbreviations

CTS: Carpal tunnel syndrome
HRQL: Health-related quality of life
SF-36: Medical outcomes short-form 36 (SF-36)
SOC: Sense of coherence.
